# Emotions alter muscle proprioceptive coding of movements in humans

**DOI:** 10.1038/s41598-017-08721-4

**Published:** 2017-08-16

**Authors:** Rochelle Ackerley, Jean-Marc Aimonetti, Edith Ribot-Ciscar

**Affiliations:** 1 Aix Marseille Univ, CNRS, LNIA, FR3C Marseille, France; 2 Department of Physiology, University of Gothenburg, 40530 Göteborg, Sweden

## Abstract

Emotions can evoke strong reactions that have profound influences, from gross changes in our internal environment to small fluctuations in facial muscles, and reveal our feelings overtly. Muscles contain proprioceptive afferents, informing us about our movements and regulating motor activities. Their firing reflects changes in muscle length, yet their sensitivity can be modified by the fusimotor system, as found in animals. In humans, the sensitivity of muscle afferents is modulated by cognitive processes, such as attention; however, it is unknown if emotional processes can modulate muscle feedback. Presently, we explored whether muscle afferent sensitivity adapts to the emotional situation. We recorded from single muscle afferents in the leg, using microneurography, and moved the ankle joint of participants, while they listened to evocative classical music to induce sad, neutral, or happy emotions, or sat passively (no music). We further monitored their physiological responses using skin conductance, heart rate, and electromyography measures. We found that muscle afferent firing was modified by the emotional context, especially for sad emotions, where the muscle spindle dynamic response increased. We suggest that this allows us to prime movements, where the emotional state prepares the body for consequent behaviour-appropriate reactions.

## Introduction

Emotions are responsible for profound changes in the body landscape, by affecting the internal environment, viscera, and the musculoskeletal system. Five basic emotions have been identified: sadness, happiness, fear, anger, and disgust^1^, where emotional reactions are displayed to the world through our behaviour, actions and reactions. Accordingly, the musculoskeletal system allows us to carry out these activities, interact with others, and explore our surroundings. It also informs us about changes in such activity, our posture, and the speed and quality of movements^2, 3^. Our behaviour influences social interactions, where even the smallest of facial muscle contractions can signal our intentions and affective thoughts^4^.

Emotions modulate our readiness to move^5^, where pleasant experiences prime approach actions and unpleasant experiences prime withdrawal. The temporal kinematics of movement are especially affected by the situational valence^6^. Emotions, particularly unpleasant ones, can impact movements, for example, producing differences in the maintenance of an isometric contraction^7^ and of posture^8^. The continued exposure to such stimuli can magnify the force of sustained voluntary movements^7^. Manipulating emotional state can also be used to facilitate gait initiation in healthy people^9^, as well as in patients such as those with Parkinson’s disease^10^.

The knowledge and control of action is determined, at least partly, by proprioceptive information, which arises from muscle spindles^11^. This is corroborated by studies on patients lacking myelinated fibres (including those for proprioception), who are unable to maintain a steady joint angle without vision, or adapt their movements to unexpected loads^12, 13^. These patients show deficits in multi-joint movements and their limb trajectories are severely distorted, even when they have visual input during movements^14^. The sensitivity of muscle afferents can be modified by the central nervous system through the gamma fusimotor system, as found in animals^15, 16^. This effect has been sought in humans, yet studies have shown only a small impact of the descending drive on muscle afferent activity^17–19^, although it has been observed recently, where muscle afferent firing acts as a forward sensory model and predicts the future kinematic activity of the parent muscle^20^.

Conscious cognitive processes have been shown to influence muscle afferent activity, where attention^21, 22^ and learning^23^ can modify their sensitivity, allowing the selection of specific muscle feedback relevant for the behavioural context. However, it is unknown whether instinctual processes, such as emotions, can modulate muscle feedback directly. We hypothesise that emotions can modify the descending motor drive and modulate the firing of muscle afferents, where emotions will prepare the body for making consequent behaviour-appropriate reactions.

## Results

**Figure 1:**
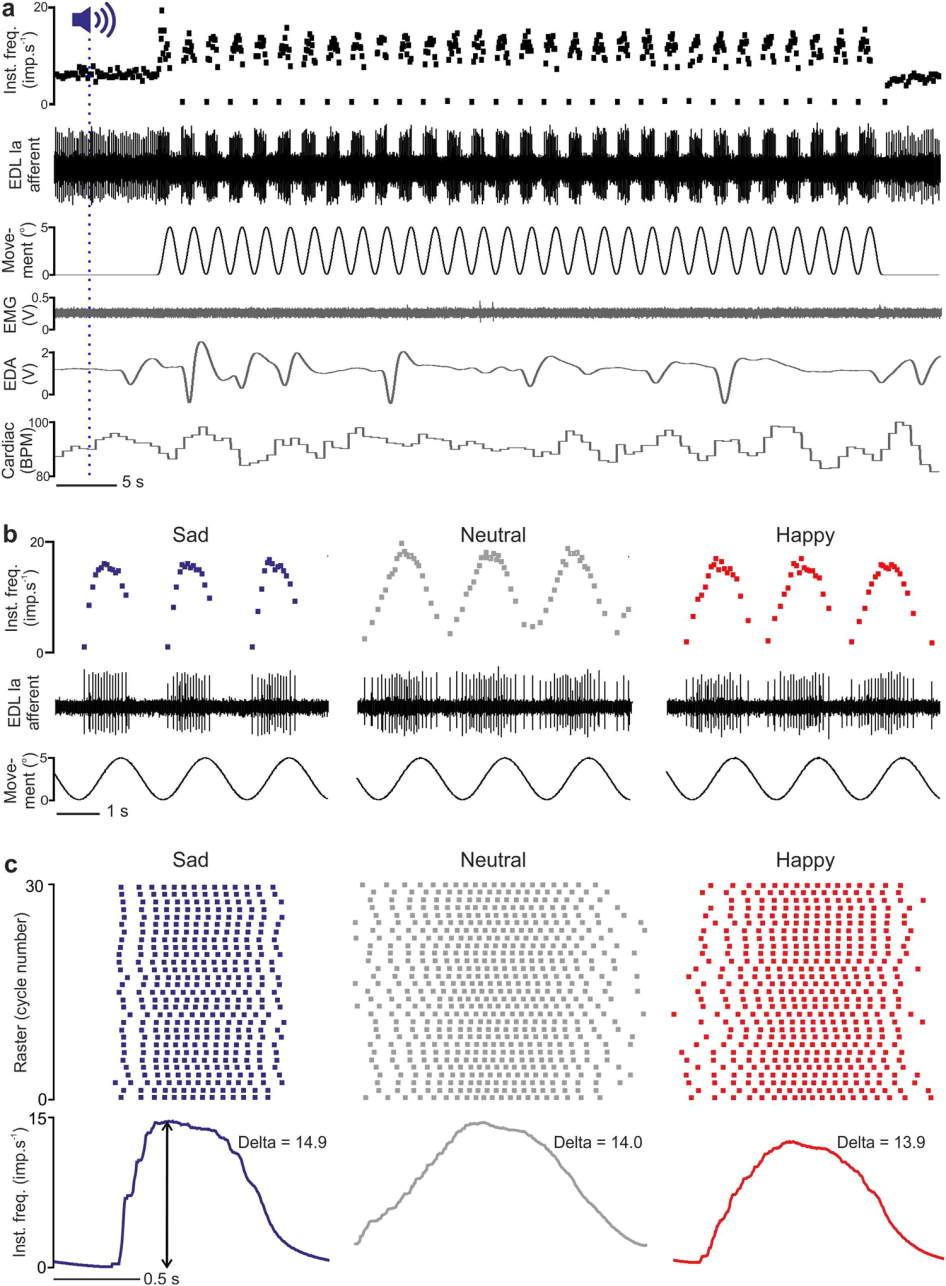
Responses from a single muscle afferent (from extensor digitorum longus, EDL) to movements during different emotional music. (**a**) Recordings to a full movement sequence during sad music. The top trace shows the instantaneous frequency of muscle afferent firing, with its activity shown below. The third trace shows the imposed sinusoidal movement, with a lack of concomitant EMG activity below. The fifth trace shows fluctuations in the electrodermal activity and the below trace demonstrates cardiac frequency. (**b**) For the same unit, activity is shown during three movements (bottom trace), over sad, neutral, and happy conditions. Differences can be seen in the instantaneous frequency (top) and unit firing (middle) between the conditions, with decreased activity during muscle shortenings in the sad condition. (**c**) Raster plots of spike activity for each condition are shown for the full movement, per sinusoid, with the mean response below. The dynamic response was measured by delta (change in the instantaneous frequency curve) for each condition.

We recorded unitary activity from 24 primary (type Ia) muscle afferents, originating in the dorsal flexor ankle muscles, using microneurography^24, 25^ in 16 resting, healthy participants. The sensitivity of each muscle afferent was assessed during an emotional manipulation paradigm, where the participant closed their eyes and listened to evocative classical music of sad, neutral, or happy valence^26–31^ (Table 1), or sat passively (no music). A microelectrode was inserted into the common peroneal nerve and the absence of concomitant muscle activity was controlled by recording surface electromyographic activity (EMG). Muscle afferent firing was recorded during sinusoidal plantar flexion/dorsiflexion movements (Fig. 1), imposed at the ankle, while the participant attended to the emotions triggered by each type of music. Electrodermal activity and heart rate were also recorded as emotional markers and participants rated their emotion on a visual analogue scale (VAS), from sad to happy.

**Table 1:** Classical music pieces selected for the experiments to induce different emotional states.

Musical piece	Composer	Valence	Number of presentations	Rating (mean ± SD)
Nocturnes, Op. 27 No. 1	Chopin	Sad	7	1.8 ± 0.2
Kol Nidrei, Op. 47	Bruch	Sad	7	2.1 ± 0.5
Adagio for Strings	Barber	Sad	5	0.4 ± 0.1
Nocturnes, Op. 48 No. 1	Chopin	Sad	2	0.8 ± 0.5
Piano Concerto No. 23, Adagio	Mozart	Sad	1	1.2
Symphony No. 5, Adagietto	Malher	Sad	1	2.4
Peer Gynt: Solveig’s song, Op. 55 No. 2	Grieg	Sad	1	3.8
The Planets, Venus	Host	Neutral	10	4.9 ± 0.4
Pictures at an exposition No. 1	Mussorgsky	Neutral	7	5.7 ± 0.3
Water music suite No. 1 Minuet (mvt 6)	Handel	Neutral	5	4.9 ± 0.2
Water music suite No. 2 passepied (mvt 2)	Handel	Neutral	1	5.9
Piano Sonata No. 14, Moonlight Sonata	Beethoven	Neutral	1	5.5
Radetzky marsch, Op. 228	Strauss	Happy	5	9.3 ± 0.3
Carmen Suite No. 1, Les Toreadors	Bizet	Happy	5	8.4 ± 0.4
Piano Concerto No. 23, Allegro assai	Mozart	Happy	5	8.2 ± 0.4
Carnival of the Animals No. 14, Finale	Saint-Saëns	Happy	3	9.0 ± 0.7
Eine kleine Nachtmusik, K. 525	Mozart	Happy	3	8.7 ± 0.4
Four seasons, Spring, Allegro	Vivaldi	Happy	2	7.9 ± 0.2
Carnival of the Animals No. 10, Aviary	Saint-Saëns	Happy	1	8.0

The musical piece, composer, its pre-determined emotional valence^26–31^, the number of presentations per piece for the whole experimental group, and the VAS mean ratings are given.

We find that muscle afferent activity was modulated by the emotional content. Figure 1 details a full example of responses from a single primary muscle afferent, while listening to the music of different valences. During sad music, the afferent response composed a succession of spike bursts and silences, relating to the lengthening and shortening of the muscle, respectively (Fig. 1a shows a full movement sequence, with all the physiological measures and Fig. 1b left shows three successive movements on an expanded time-scale). The same afferent’s response to movement during neutral music differed considerably (Fig. 1b, middle), where the silent periods between sinusoids disappeared and there was additional firing throughout these parts of the movement cycle. When listening to happy music, silences occurred during muscle shortenings, akin to the sad condition, but these were shorter in duration (Fig. 1b, right). Movement-by-movement rasters (Fig. 1c) show that the afferent’s response was consistent over the movement cycles, for each condition. The mean instantaneous frequency is shown below the rasters, where the minimum and maximum rates were extracted. The difference between these two measures (‘delta’) was used to quantify the afferent’s dynamic firing and we found increases in the sad condition, as compared to the neutral and happy conditions.

**Figure 2:**
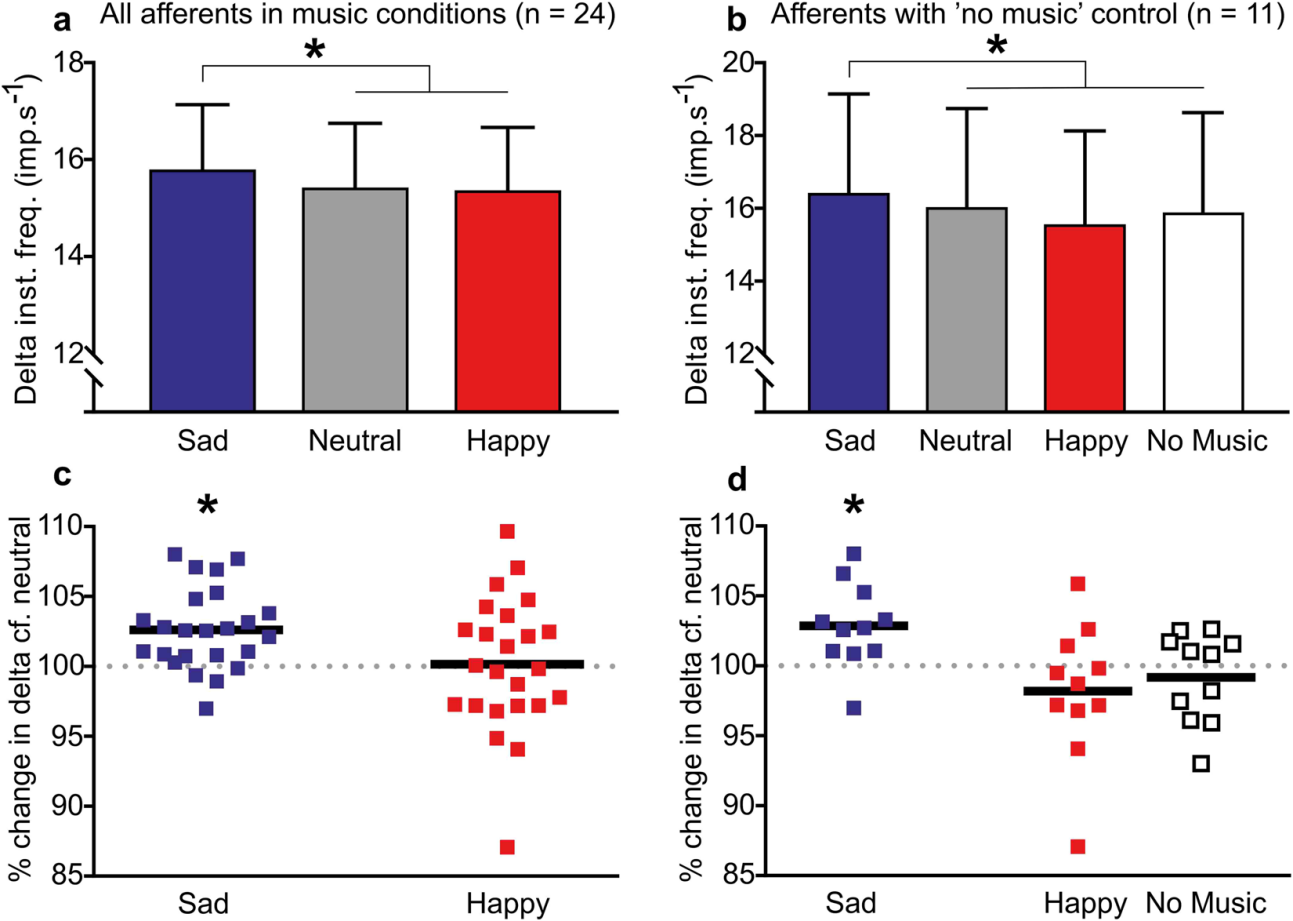
Modulation of the muscle afferent dynamic responses over the conditions for the full group of units (left) and a sub-set with the additional no music condition (right). The top graphs show the overall changes in instantaneous firing frequency (delta). (**a**) A significant increase in delta was found during the sad condition, over the neutral and happy conditions for all units, which was also found in (**b**) the sub-set with the additional no music condition. Means with SEM are shown. The bottom row shows the spread of the population. (**c**) The distribution of delta (as a percentage) in comparison to the neutral music condition for all units. (**d**) The same distributions are shown for the sub-set of units with the no music condition. In both (**c**) and (**d**), the delta in the sad condition was significantly increased. *p < 0.05.

For the whole population of afferents (n = 24), the delta significantly differed between the music conditions (ANOVA, F_(2,46)_ = 4.28, p = 0.034, η^2^ = 0.16). Figure 2a shows that the delta was significantly higher during sad music, as compared to happy music (p = 0.031) and neutral music (p = 0.001), but did not change significantly between happy and neutral music (p = 0.812). Figure 2c shows the distribution of responses in the emotional conditions, as compared to neutral. Here, an increase in delta during the sad condition was found in 21/24 units, where the delta during sad music was significantly increased (t = 4.43, p < 0.001).

We also compared the changes in muscle afferent delta in a sub-population of the same units when no music was listened to, as an auditory control. This sub-set (n = 11) showed the same pattern of responses, where the delta was significantly different between the conditions (ANOVA, F_(3,30)_ = 5.21, p = 0.014, η^2^ = 0.34). Figure 2b shows that the delta during sad music was significant increased, as compared to the neutral (p = 0.023), happy (p = 0.011), and no music (p = 0.025) conditions. There was no significant difference between the neutral and no music conditions (p = 0.456). The distributions of the responses over the conditions, as compared to neutral, are shown in Fig. 2d. Here, an increase in delta during the sad condition was found in 10/11 units, where the delta during sad music was significantly higher than during neutral music (t = 3.15, p = 0.010).

**Figure 3:**
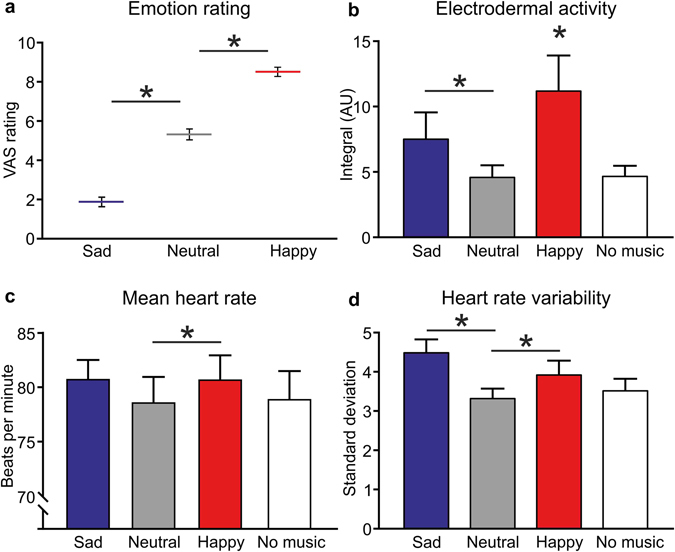
Changes in emotional ratings and physiological states over the conditions. (**a**) VAS ratings in the sad-happy dimension are shown over the emotional music conditions tested in the microneurography experiment. There was a significant difference between all the conditions. Physiological measures over all the experimental conditions (sad/happy/neutral music and no music). (**b**) Electrodermal activity was significantly increased during happy music, and there was a significant difference between sad and neutral music. (**c**) The mean heart rate was significantly higher during happy, as compared to neutral music. (**d**) Heart rate variability also changed with sad and happy emotions; a significant increase was found over the neutral music, and additionally, the sad emotion condition was significantly higher than the no music condition (not indicated for clarity). Means are shown with SEM, *p < 0.05.

For the physiological measures of emotion, electrodermal activity increased significantly during sad music, as compared to neutral music (Wilcoxon, W = −166 p = 0.046). In the happy music condition, the electrodermal activity was significantly higher than in all the other conditions (cf. sad W = −193, p = 0.019; neutral W = −324, p < 0.001; no music W = −208, p = 0.004; Fig. 3b). The mean heart rate increased significantly during happy music, as compared to neutral music (W = −139, p = 0.046; Fig. 3c). Heart rate variability was significantly higher during sad music (W = −169, p = 0.008) and happy music (W = −78, p = 0.043; Fig. 3d), as compared to neutral. Heart rate variability was also increased during sad music, as compared to no music (W = −134, p = 0.010). These measures were congruent with the emotion VAS ratings; a significant effect was found between the emotional conditions (Friedman ANOVA = 41.54, p < 0.001), where there were differences between all the comparisons (all p < 0.01; Fig. 3a). This showed that participants felt sad, neutral, and happy emotions when listening to the corresponding music.

## Discussion

The present results demonstrate that changes in emotional state lead to the differential encoding of body movement by muscle spindles. We used the auditory modality to provoke emotional changes, as the induced affect is more pervasive compared to other modalities^31^. The emotional content of the music was sufficient to drive central mechanisms and modulate the descending fusimotor efference to muscles, as seen in the muscle afferent responses, as well as produce physiological changes.

We found that sad emotions caused muscle afferents to respond differently to movement, as compared to the effects from neutral music (in 20/24 units), as seen in an increase in their dynamic firing. The congruence between the sad feelings induced by listening to sad music and those triggered by the experimental situation (e.g. not being able to move), likely amplified the effect^32^. Sad feelings may be more important for survival^33^ and prime the body for a context-appropriate behavioural response e.g. withdrawal and avoidance. Unpleasant stimuli generally provoke a more pervasive behavioural and physiological reaction^5–7, 31, 34–36^, where corticospinal excitability has been found to be higher for unpleasant stimuli^5, 37–40^, reflecting increased motor preparation. Such studies demonstrate only small modulations in corticospinal excitation by emotions; these changes may not produce an actual movement, yet they will nevertheless influence the execution of future movements^39^. Presently, we found a small, but consistent increase in the dynamic firing of muscle afferents during sad emotions, which also suggests a change in the readiness to react.

Our findings during happy emotions were more variable, where we found both increases and decreases in the dynamic firing over different participants. The increases in dynamic firing may be akin to the sad condition, where there is priming for action; as generally observed with positive emotions that prompt approach behaviour^6^. However, we suggest that, for some participants, a situational incongruence may have occurred. The happy music may have evoked a desire to move, but opposingly, the participant was required to remain relaxed for the experiment. Previous work has postulated that pleasant stimuli trigger an urge to move, where corticospinal excitability is decreased due to a need to suppress the response^6^. Hence, the decreases in the dynamic response of muscle afferents that we found during happy music may reflect a suppression of the urge to move. Thus, the participant’s perception of the situational incongruence may have dictated their overall response, demonstrating the subjective nature of emotional experiences and reactions^41^.

Muscle proprioceptive messages encode both the position and velocity of movements^2^, and the present increase in their dynamic firing likely leads to increased proprioceptive acuity during emotional situations, particularly sad ones that may require more urgent attention^33^. The change in muscle afferent firing that we demonstrate with emotion is not large; however, muscle afferent firing rates are much lower in humans, as compared to animals^21^. For example, the fusimotor-induced increase in muscle afferent firing rate during isometric contractions in humans (~20 Hz)^42^ is considerably less than that in awake cats (100–300 Hz)^43^. At these lower frequencies, the difference in afferent firing will be less pronounced in humans^23^, as we find. Nevertheless, the muscle afference sent to the brain will be registered and enhance ongoing behaviour, preparing the person for behaviour appropriate to the situation. We are not saying that a subsequent behaviour must necessarily be produced; rather that the emotional response readies the body for action to suit the situation and muscle afferent signals could act as ‘somatic markers’ in driving behavior^44^.

Muscle afferents are involved in the regulation of motor activities^45^, where an increase in their dynamic firing may influence movements by directly enhancing alpha motoneuron excitability^46^. Similarly, a descending command appears to govern increases in sympathetic activity to muscles, rather than direct muscle activity, which is important for the regulation of muscle perfusion during movement^47^. Regarding the origin of the effect we find, it is improbable that the increase in muscle spindle sensitivity resulted from enhanced muscle sympathetic activity, since it does not influence muscle spindle firing^48^. The described effect is likely produced by the descending drive from fusimotor neurons, and more specifically, from the dynamic gamma fusimotor drive that increases the tension in intrafusal muscle fibres^15, 16, 49, 50^.

As well as the dynamic change in muscle afferent firing, we found differences in the physiological measures over the conditions. We expected electrodermal activity to increase in emotional states, as studies have found skin conductance changes when listening to emotional music, and akin to our findings, that happy emotions produce a larger effect^34–36^. Similarly, our emotional conditions produced changes in the heart rate measures, as found previously^31, 34^. These measures reflect the effect of feeling the emotions in the music, as neutral music did not show such an impact and the physiological reactions from it were equivalent to the no music condition.

Emotions are vital to human behaviour and for social interactions. They shape our perceptions and actions, particularly through the auditory and visual channels^29^. We find that emotions modulate the feedback from our muscles, which has direct consequences for our bodily awareness and readiness to react to emotional situations, i.e. perception-action. The effect of emotions on driving preferential action responses may be exploited therapeutically. The addition of external stochastic noise has been shown to reduce physiological tremor and improve motor performance^51^, as well as postural control^52^, and the manipulation of internal emotional state may ready the body for action. Music is widely used to enhance emotional impact and emotional priming may be used in physical rehabilitation to enhance feedback in patients with movement disorders^10^. Likewise, emotional manipulation may be used in patients with affective disorders, such as depression, where listening to sad music may actually regulate mood and behavior^53^.

## Methods

### Participants and experimental set-up

The experiments were performed on 16 healthy human volunteers (6 females, mean age 27.7 ± 6.3 years), all of whom gave their written, informed consent. The study was approved by the local ethics committee (Comité de Protection des Personnes Sud-Méditerranée I) and performed in accordance with the Declaration of Helsinki. Due to the nature of the paradigm (*in vivo* peripheral nerve single unit recordings during an emotional manipulation), it was important that all our participants were calm and felt relaxed about the procedure. Hence, they participated in a pre-experiment visit to assess their suitability and introduce them to the laboratory environment. For the experiment, participants were seated comfortably in an armchair with their legs positioned in cushioned grooves. This allowed a standardised, relaxed position to be maintained, in the absence of any muscle activity. The knee joints were positioned at an angle of ~120–130°, and their feet rested on supports, giving a neutral position of the ankle, with an angle of ~110° between the leg and the foot. The right foot was laid on a stationary plate and the left foot was placed on a pedal connected to a computer-controlled robot, which was used to impose plantarflexion/dorsiflexion movements.

Before the experiment started, the participant listened to several pieces of classical music deemed to evoke sad, neutral, or happy emotions (up to 5 pieces of each type), and rated each of them on a VAS, ranging from sad (equating to 0, the saddest they could feel) to happy (equating to 10, the happiest they could feel). Although emotions are complex, we chose sad and happy as the emotions to be induced, as these are two of the five basic emotions^1^, where sad-happy can be represented as a dimension on a continuum. Sad and happy emotions can be readily induced experimentally and have been well-studied, including comparing sad, neutral, and happy emotions through images^54–56^ and music^26, 27, 30, 31, 57^. We used the auditory modality to invoked these emotions, as the effect is more pervasive compared to other modalities^31^. For classical music, sad and happy emotional music has been well-classified^26, 27^, and produces body physiological changes^34–36^, evokes neural activity in brain regions relating to emotional processing^27^, and induces autonomic responses^31^. Further, even when pleasure can be derived from sadness (e.g. enjoying sad music), the resultant emotion is decoupled from the aesthetic content (liking) of a stimulus^53, 58^. As our approach introduced constraints on the number of conditions presented (e.g. recording stably from a single muscle afferent), we focused on sad-happy as our emotional dimension, with neutral music and no music as emotional and auditory controls, respectively.

For each participant, the most effective music in triggering each type of emotion, for sad, neutral, and happy pieces, was chosen to be played during physiological recordings. Table 1 gives the name of each piece of music and the number of times the music was used, with the mean emotional ratings from all participants. The musical pieces were subject to signal processing to provide similar levels of acoustic excitation and to avoid strong variations of the sound level. Original music digital recordings in MP3PRO (FHG) format (44100 Hz, 16 bits) were first adjusted to have similar root mean square amplitudes of 80 dB re. 1 bit, using a custom made MATLAB (The Mathworks, Natick, MA) program. An amplitude compression was then performed using Adobe Audition 1.5 (Adobe, San Jose, CA) software. The chosen compression curve flattened the upper 40 dB (all levels from maximum to −40 dB re. maximum) to a constant level. A linear compression was then applied to the lower levels (all levels from −40 dB re. maximum to minimum). These modifications of the music were not particularly noticeable to the participants, but ensured equivalent modulations between the music pieces. The participants rated these music pieces easily for their emotional content (see Fig. 3a and Table 1).

### Physiological recordings

The activity of single muscle spindle endings, originating from the tibialis anterior (TA; n = 10) and extensor digitorum longus (EDL; n = 14) muscles, were recorded from the left common peroneal nerve at the popliteal fossa, using the human, *in vivo* technique of microneurography^24, 25^. We ensured that the procedure was not painful and that the participant remained calm and relaxed throughout (e.g. we did not use electrical stimulation to find the nerve, we advanced the electrode very slowly and carefully, we frequently asked them how they were, and monitored their physiological responses). Unitary muscle afferent activity was recorded referentially using an insulated tungsten microelectrode (impedance 0.3–1 MΩ, tip diameter ~5 µm, length 30 mm; FHC, Bowdoin, ME). The recordings were monitored continuously using an oscilloscope and a loudspeaker. Neural activity was amplified (×200,000) and band-pass filtered (300–3000 Hz) to ensure an optimal signal-to-noise ratio, and sampled at 20 kHz frequency. Muscle afferents were identified as primary endings on the basis of their irregular spontaneous activity, their high dynamic sensitivity to ramp and hold movements, and their silencing during passive muscle shortenings^59^.

The absence of concomitant muscle activity was controlled throughout the experiment by recording EMG. Two pairs of surface electrodes were placed over the TA muscle, with an inter-electrode distance of ~4 cm for each pair. The EMGs were recorded with high gain (×10,000), band-pass filtered (30–300 Hz), and sampled at 5 kHz. Further measures of emotional responses were obtained through recording electrodermal activity, using two surface electrodes placed on each side of the left hand (gain: ×500, band-pass: 0.1–100 Hz, sampling frequency: 200 Hz), and heart rate was recorded by means of a passive transducer, strapped around the left index finger. The EMG, electrodermal activity, and heart rate measures were monitored throughout the experiment. These provided feedback to the experimenter that the participant was not stressed, felt trapped, or wanted to move (e.g. no EMG, not a high heart rate, no strong modulations in the electrodermal response).

### Experimental paradigm

The participant’s emotional state was changed by listening to classical music through headphones. The music pieces had been previously defined as reliably inducing sad (n = 9 pieces), happy (n = 7), or neutral (n = 5) affective states^26–31^ (see Table 1). Once activity from a single muscle spindle had been isolated, the participant put on the headphones and was instructed to close their eyes. The participant was told before the recording to listen attentively to the music and disregard the movement imposed at the foot. The music started and, after a delay of ~10 s, a series of 30 sinusoidal plantarflexion/dorsiflexion movements (5° amplitude and 5°/s velocity, over ~1 minute) were imposed at the ankle joint. The movement range was chosen to maintain stability of the microelectrode recording at the level of the knee, but allow sufficient ankle movement^21^. The maximal angle towards plantar flexion is ~20°^60^, hence 5° amplitude corresponds to ~25% of the maximal movement possible.

We chose to play 10 s of music before the movements started to allow the participant to ‘feel’ the emotions. Music has been found to be instantly evocative, where the physiological arousal changes quickly from the very beginning of the music^35, 61^. The initial period allowed sufficient time for continued experience of the emotions during the movement, without inattention or habituation^31, 34^. Each afferent was recorded during four sequences, where the participant listened to sad, happy, or neutral music, or listened to nothing (‘no music’ condition) through noise-cancelling headphones (Bose; Framingham, MA). These four conditions were presented in a pseudo-random order. After each sequence, the participant was asked to rate the emotion felt for the music on a further VAS, which was used in the analysis.

### Data analysis and statistics

The data were stored on a digital tape recorder (DTR 1802, Biologic, Claix, France) and processed off-line using Spike 2 software (CED, Cambridge, UK). The nerve spikes were inspected carefully in an expanded time scale and transformed into an instantaneous frequency curve. The mean instantaneous frequency curve (bin size = 0.005 s), per unit/condition, was obtained by averaging its response to 29 sinusoidal movements, where the first movement was excluded because of a dynamic response from the onset of the movement. The maximum and minimum frequency was extracted from this measure, and the difference (‘delta’; see Fig. 1c) was used as an index to characterise a unit’s dynamic response in each condition. This measure was chosen specifically to reflect the dynamic response of muscle afferents, where similar measures have been used previously^62^. Note that in the majority of microneurography studies investigating changes in muscle spindle activity use ramp-and-hold movements. In our paradigm, we needed to use a longer-lasting movement stimulus to determine the effects of emotions (which can be induced quickly, but fluctuate over time), hence we were required to deliver continuous movements over time. We measure the changes in firing rate in cycle histograms over the sinusoids, as in previous animal studies dealing with the effect of fusimotor activity^63, 64^. For six of the units, muscle activity (EMG) was present during some of the movement cycles; in these cases the averaged response was composed of the movements that were EMG-free.

The statistical analysis for the whole population of afferent responses was made using a one-way ANOVA with repeated measures, followed by false-discovery rate adjusted post-hoc tests. A second ANOVA was used to compare the data in a sub-set of units, where the no music condition was also tested. The effect sizes were determined using Eta-squared (η^2^). The emotional VAS ratings were compared using a non-parametric Friedman’s ANOVA and differences between the music conditions were compared using Dunn’s corrected post-hoc tests. The physiological measures (electrodermal activity, mean and standard deviation of heart rate) were recorded over all the conditions (including the no music condition) and non-parametric Wilcoxon tests were used to compare differences within each measure, due to the data not meeting normality criteria (which was found using Shapiro-Wilk tests). The level of significance was set at p < 0.05.

### Data Availability

The datasets generated during and/or analysed during the current study are available from the corresponding author on reasonable request.
